# Dopamine D2 Receptors and Its Downstream Signaling in Compulsive Eating

**DOI:** 10.3390/brainsci15090923

**Published:** 2025-08-27

**Authors:** Caden Leung, Kabirullah Lutfy

**Affiliations:** 1 Diamond Bar High School, 21400 Pathfinder Road, Diamond Bar, CA 91765, USA; cadenleungca@gmail.com; 2 Department of Biotechnology and Pharmaceutical Sciences, College of Pharmacy, Western University of Health Sciences, 309 East 2nd Street, Pomona, CA 91766, USA

**Keywords:** food reward, dopamine D2 receptor, compulsive eating, high-fat diet

## Abstract

Obesity has become a major public health crisis and serves as an underlying condition for other chronic metabolic diseases. The dysregulation of the inhibitory and regulatory mechanisms of the mesolimbic dopamine system, particularly dopamine D2 receptors (D2Rs), plays a critical role in driving excessive food consumption and compulsive eating habits. Based on the current literature, chronic consumption of high-fat foods elicits hedonic sensations and has the potential to downregulate and desensitize D2Rs, impairing their signaling and inhibitory action. This impairment thereby alters the downstream signaling of the D2Rs, involving the inhibition of adenylyl cyclase and the associated cascade. Although individual components of this proposed pathway have been studied, a comprehensive synthesis has not been established. This review aims to explore the relationship between D2R downregulation and desensitization and their effects on the downstream signaling cascade. We hypothesize that alterations in this pathway may lead to the dysregulation of the expression of orexigenic and anorexigenic neuropeptides, contributing to binge-eating behaviors.

## 1. Introduction

Recently, obesity has emerged as a prevalent public health issue, defined as the excess accumulation of body weight and body fat. This underlying condition continues to serve as a breeding ground for other chronic health issues such as type 2 diabetes mellitus and cardiovascular diseases. Food intake plays an important role in obesity. The consumption of certain foods modulates mechanisms that regulate body weight and neural pathways that control satiety and food reward. In particular, high-fat foods have been shown to elicit increased food reward through the activation of the mesolimbic pathway. This heightened food reward can lead to increased caloric intake and, ultimately, compulsive eating behaviors.

The relationship between food intake, food reward, compulsive eating, and obesity lies at the core of the mesolimbic dopaminergic pathway, which originates in the ventral tegmental area (VTA) and projects to the nucleus accumbens (NAc). When stimulated by palatable food containing high fat, dopamine is released in the NAc [1], promoting pleasure and satisfaction. These hedonic responses are mediated by dopamine D2 receptors (D2Rs), which serve as an essential negative feedback mechanism to prevent overstimulation by regulating dopamine release. Over time, however, chronic consumption of high-fat foods can dysregulate this regulatory mechanism via dopamine signaling. Indeed, previous research has shown that repeated exposure to such stimuli impairs the inhibitory function of the D2Rs [1].

Although the relationship between obesity, D2Rs, and food intake has been extensively studied in general, the downstream signaling effects of desensitized and downregulated D2Rs in hedonic regulation remain unclear. This review aims to unify findings on D2R dysregulation with downstream intracellular changes, particularly within the cAMP/PKA/pCREB pathway, to provide a conceptual model of how these mechanisms contribute to compulsive eating behaviors.

### 1.1. Literature Search and Key Words

This manuscript reviews existing literature and studies from PubMed and Google Scholar databases, including systematic reviews, expert opinions, human neuroimaging studies, and primary experimental rodent studies. Searches combined terms such as D2R, high-fat, NPY, AgRP, POMC, GPCR, CART, orexigenic, anorexigenic, food reward, compulsive eating, cAMP, CREB, pCREB, leptin, and ghrelin. The inclusion criteria were peer-reviewed articles published between 1989 and 2025 written in English that examined food consumption, D2R function and regulation, cAMP/PKA/CREB signaling, neuropeptide modulation, and energy homeostasis in humans or rodents. Exclusion criteria included articles published prior to 1989, articles written in languages other than English, studies irrelevant to these topics, or those focused solely on dopamine receptors other than D2Rs. Selected articles were analyzed for relevance to the review objectives.

### 1.2. Homeostatic Regulation of Food Intake

The body is composed of a series of complex regulatory interactions and mechanisms that help maintain energy homeostasis and ensure that caloric intake aligns with energy expenditure. The hypothalamus is crucial for this regulation, as it integrates metabolic signals controlling hunger and satiety. Ghrelin, secreted primarily by the stomach, stimulates appetite, while leptin, secreted by adipose tissue, suppresses appetite by signaling that energy reserves are sufficient [2].

In addition to these hormones, several neuropeptides in the hypothalamus, notably neuropeptide Y (NPY), agouti-related peptide (AgRP), pro-opiomelanocortin (POMC), and cocaine-and-amphetamine-regulated transcript (CART), also play critical roles in food intake and satiety. NPY and AgRP are orexigenic neuropeptides that stimulate appetite and food intake, a process that is crucial in energy reserve deficits [2]. The expression of these orexigenic neuropeptides is often upregulated during periods of fasting or caloric restriction. In contrast, POMC and CART are anorexigenic neuropeptides that suppress caloric intake when energy reserves are sufficient and promote fullness and energy expenditure in response to leptin [3]. Together, the interactions between these hormones and neuropeptides, both orexigenic and anorexigenic, create a dynamic feedback loop that helps mediate food intake.

### 1.3. Hedonic Regulation of Food Intake

While food intake is regulated by homeostatic mechanisms for energy and caloric balance, eating is also driven by pleasure and reward. This phenomenon, termed hedonic eating, describes food consumption for pleasure. Palatable foods rich in fat and carbohydrates are particularly known to stimulate the brain’s mesolimbic dopamine system [4]. While this review emphasizes the role of high-fat diets, similar effects are observed with high-sugar diets, indicating a broader role of palatability in D2R-mediated reward [4]. Unlike homeostatic food intake, which is driven by hunger and satiety signals from ghrelin and leptin, respectively, hedonic food consumption is fueled by external factors, emotional factors, and/or cognitive cravings despite sufficient energy reserves, which may lead to overconsumption [5]. When individuals engage in excessive consumption of these foods, these hedonic sensations may override the existing regulatory and inhibitory mechanisms of the mesolimbic dopamine system.

### 1.4. D2R-Mediated Regulation of Food Intake Under a Physiological State

**Figure 1:**
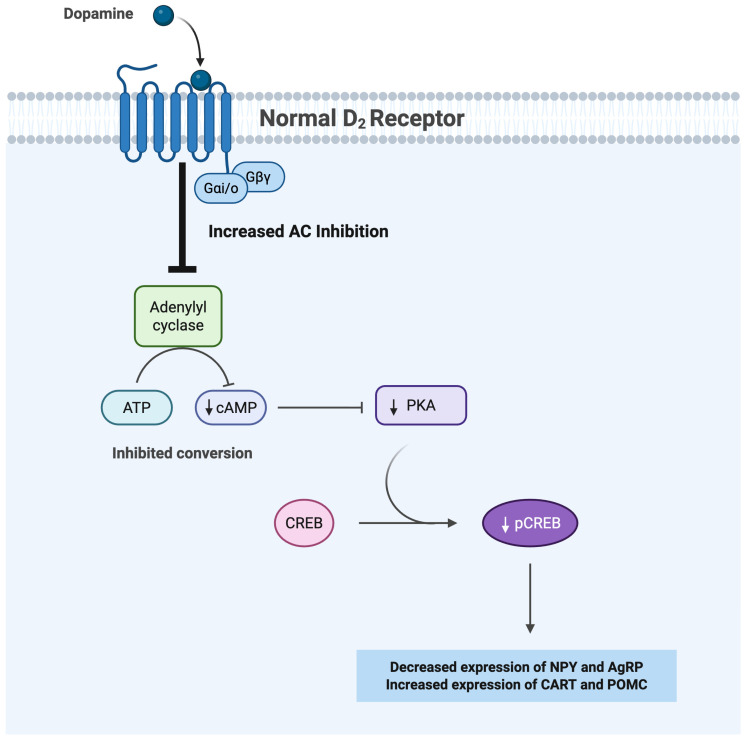
A schematic diagram of the consequences of D2R activation on orexigenic and anorexigenic peptides in the hypothalamus. D2R activation inhibits AC activity (shown by the thick inhibitory arrow), lowering cAMP and PKA signaling. This reduces pCREB (shown by a downward arrow), thereby altering gene expression, leading to the downregulation of orexigenic neuropeptides (NPY, AgRP), and the upregulation of anorexigenic neuropeptides (POMC, CART). Abbreviations: AC, adenylyl cyclase; cAMP, cyclic adenosine monophosphate; PKA, protein kinase A; pCREB, phosphorylated cAMP-response element binding protein; NPY, neuropeptide Y; AgRP, agouti-related peptide; POMC, pro-opiomelanocortin; CART, cocaine-and-amphetamine-regulated transcript; VTA, ventral tegmental area; NAc, nucleus accumbens.

Dopamine D2 receptors are G-protein-coupled receptors that play a critical role in regulating food reward and inhibitory responses within the mesolimbic dopaminergic neurons through Gi/o protein signaling. These receptors are located in various areas in the brain, most notably the striatum, NAc, VTA, and hypothalamus [6,7,8,9]. When dopamine binds to D2Rs on the presynaptic dopaminergic neurons, this interaction serves as a negative feedback mechanism leading to a decrease in the release of dopamine and the reward associated with food intake [10]. Moreover, in individuals with normal D2R function, dopamine binds to the D2R, leading to the inhibition of adenylyl cyclase (AC), an enzyme responsible for the conversion of adenosine triphosphate (ATP) to cyclic adenosine monophosphate (cAMP) [11]. Likewise, a study investigating dopamine’s effects on osteoclastogenesis reported that the binding of D2R inhibits the cAMP/protein kinase A (PKA) pathway, which ultimately decreases phosphorylated cAMP-response element binding protein (pCREB) levels [12]. Additional studies have also reported a positive relationship between the quantity of pCREB and the expression of orexigenic peptides, such as NPY and AgRP, both of which promote food intake and stimulate appetite [13,14,15,16,17]. As shown below (Figure 1), a decrease in pCREB not only lowers the expression of orexigenic neuropeptides, but also increases anorexigenic potential, defined as the ratio of mRNA abundance of anorexigenic neuropeptides to that of the orexigenic neuropeptides [18,19]. It is important to note that besides its role as a transcription factor, pCREB also serves as an epigenetic regulator. Phosphorylation of CREB promotes the recruitment of CREB binding protein (CBP) and p300 co-activators, histone acetyltransferases that increase promoter accessibility and transcription of neuropeptide genes through histone acetylation [20,21,22,23].

Under normal physiological conditions, the D2R/cAMP/PKA/pCREB candidate signaling pathway functions to regulate feeding behaviors and reward. However, chronic exposure to high-fat foods impairs D2R sensitivity and reduces D2R expression, compromising the integrity of dopamine-mediated control of inhibitory signaling, and leading to binge-eating behaviors.

### 1.5. D2R-Mediated Regulation of Food Intake Under a Downregulated/Desensitized State

Over time, the intake of high-fat foods can dysregulate the hedonic regulation of the dopamine system. One characteristic of those who repeatedly consume high-fat foods is the decreased expression of D2Rs, termed D2R downregulation, and/or D2R desensitization [10,24,25]. Previous studies have shown that D2Rs can undergo downregulation and desensitization. In this process, receptor phosphorylation by G protein-coupled receptor kinases leads to the recruitment of β-arrestins (β-arrestin-1 and 2) that are primarily responsible for uncoupling G-protein signaling and receptor internalization via endocytosis [26,27,28]. However, the exact mechanism of how high-fat diets drive GPCR downregulation and desensitization has not been established, requiring further research. Another study showed that extended access to the cafeteria diet led to significantly higher body weight and a reduction in the membrane-bound form of D2R in the diet-induced obese rats compared to those with chow-only or restricted access [1]. Wang and colleagues used positron emission tomography to detect the availability of D2Rs demonstrated that obese individuals had reduced D2R binding, in comparison to healthy individuals with a normal body mass index [25]. Furthermore, the study by Koyama and colleagues revealed that increasing concentrations of quinpirole, a D2R agonist, greatly diminished the inhibitory response in obese mice on a 45% high-fat diet, suggesting that high-fat diet-induced obesity leads to reduced D2 receptor-mediated inhibition [10]. Taken together, these studies thereby reveal the clear inverse relationship between body weight and D2R expression levels.

This inverse association between body weight and D2R function contributes to a phenomenon termed reward hypofunctionality [1]. As a result of D2R downregulation and desensitization, individuals increase their consumption of high-fat food stimuli to fulfill the same level of food reward previously achieved. This, therefore, creates a vicious cycle that exacerbates the impairment of feedback inhibition and the dysregulation of the dopamine system, leading to addictive behaviors and abnormalities in the downstream signaling effects.

### 1.6. D2R/cAMP/PKA/pCREB Signaling Pathway in Compulsive Eating

**Figure 2:**
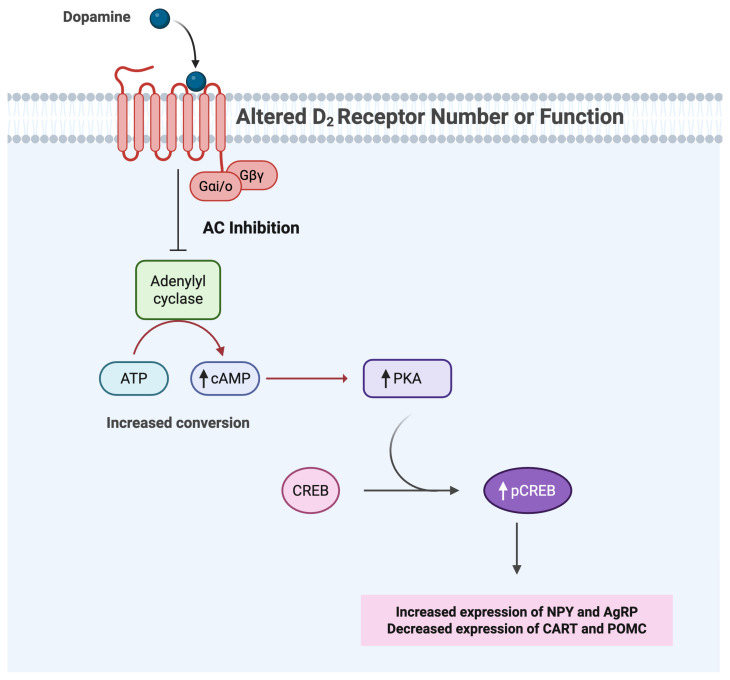
A schematic diagram of the impact of desensitized or downregulated D2R on orexigenic and anorexigenic peptides in the hypothalamus. Desensitization or downregulation of D2R reduces the inhibition of AC activity, elevating cAMP/PKA signaling and pCREB levels (shown by an upward arrow). This hypothesized shift upregulates orexigenic neuropeptides (NPY, AgRP) and downregulates anorexigenic neuropeptides (POMC, CART). Abbreviations: AC, adenylyl cyclase; cAMP, cyclic adenosine monophosphate; PKA, protein kinase A; pCREB, phosphorylated cAMP-response element binding protein; NPY, neuropeptide Y; AgRP, agouti-related peptide; POMC, pro-opiomelanocortin; CART, cocaine-and-amphetamine-regulated transcript; VTA, ventral tegmental area; NAc, nucleus accumbens.

The disrupted D2R function alters key intracellular pathways involved in modulating reward signals and can have serious implications regarding feeding behavior. Receptor desensitization and downregulation contribute to the reduced ability to transduce dopamine signals through D2Rs. As shown in studies [11,12], disruption in Gi/o protein signaling reduces AC inhibition. This increases ATP conversion to cAMP, dysregulating cAMP/PKA signaling, leading to increased phosphorylation of CREB. This altered phosphorylation of CREB, associated with D2R dysfunction, is likely to contribute to the upregulation of orexigenic neuropeptides (i.e., NPY and AgRP), and the downregulation of anorexigenic neuropeptides (i.e., CART and POMC) [13,14,15,16,17]. The additional regulation provided by pCREB through CBP and p300 recruitment for chromatin remodeling, as discussed previously, further strengthens the link between disrupted D2R signaling and altered neuropeptide expression. Thus, impaired D2R function shifts the homeostatic balance towards the expression of orexigenic neuropeptides, further driving compulsive eating behaviors (Figure 2). The altered hypothalamic neuropeptide expression influences not only hunger and satiety, but also the mesolimbic reward circuit. AgRP neurons influence the sensitivity of dopamine reward pathways; CART provides reciprocal feedback to midbrain dopamine neurons; NPY afferents link hypothalamic physiological control to VTA-driven motivation; and POMC signaling, besides its role in satiety, includes projections to the VTA that modulate dopamine excitability and motivation [29,30,31,32,33,34].

## 2. Conclusions

The findings discussed here emphasize the essential role of D2R desensitization and downregulation in regulating feeding behavior and reward signaling. With current studies elucidating only partial mechanisms of the candidate D2R/cAMP/PKA/pCREB pathway, we hypothesize that palatable high-fat foods desensitize and downregulate D2Rs, disrupting cAMP/PKA signaling by elevating cAMP levels. This promotes phosphorylation of CREB and increases the expression of orexigenic neuropeptides, ultimately exacerbating compulsive eating behaviors. Given the significant impact of disrupted D2R function on the neural circuitry modulating food reward, additional research into the molecular mechanisms and cellular processes that cause underlying receptor desensitization and downregulation, in the context of high-fat diets and obesity, is necessary to develop potential therapeutic solutions to resensitize D2Rs and restore normal D2R-mediated inhibition. The possibility of therapeutic modalities and interventions that target the restoration of D2R sensitivity and function could profoundly address the root cause of obesity and other related disorders.

### Limitations and Future Direction

Currently, most experiments and studies examining the association between D2R desensitization or downregulation, downstream signaling, and feeding behaviors have been conducted on rodent models. While these studies provide valuable insight into D2R regulation and signaling, important species differences limit direct extrapolation of the findings to humans. Further preclinical studies are needed to test the hypothesis that dysfunctional D2Rs and their downstream signaling cascade drive binge eating. Studies involving human tissues and neuroimaging will clarify how these molecular changes manifest in human populations and enhance translational relevance. Furthermore, additional pharmacological studies are required to evaluate whether D2Rs or components of the downstream signaling pathway can serve as potential therapeutic targets. Elucidating the relationship between the D2R/cAMP/PKA/pCREB pathway and feeding behavior will serve as a major advancement in our understanding of the underlying molecular mechanisms of obesity and binge-eating behaviors.

## Data Availability

No new data were created or analyzed in this study.

## Abbreviations

The following abbreviations are used in this manuscript:

AC	Adenylyl cyclase
AgRP	Agouti-related peptide
ATP	Adenosine triphosphate
cAMP	Cyclic adenosine monophosphate
CART	Cocaine-and-amphetamine-regulated transcript
CREB	cAMP-response element binding protein
D2R	Dopamine D2 receptor
NAc	Nucleus Accumbens
NPY	Neuropeptide Y
pCREB	Phosphorylated cAMP-response element binding protein
PKA	Protein kinase A
POMC	Pro-opiomelanocortin
VTA	Ventral tegmental area
